# Supplementary material for: β-actin dependent chromatin remodeling mediates compartment level changes in 3D genome architecture
Source: Nat Commun. 2021 Sep 2;12:5240. doi: 10.1038/s41467-021-25596-2 (PMC8413440; doi:10.1038/s41467-021-25596-2)
Supplement: Supplementary file 1 — Supplementary Information [file 41467_2021_25596_MOESM1_ESM.pdf]

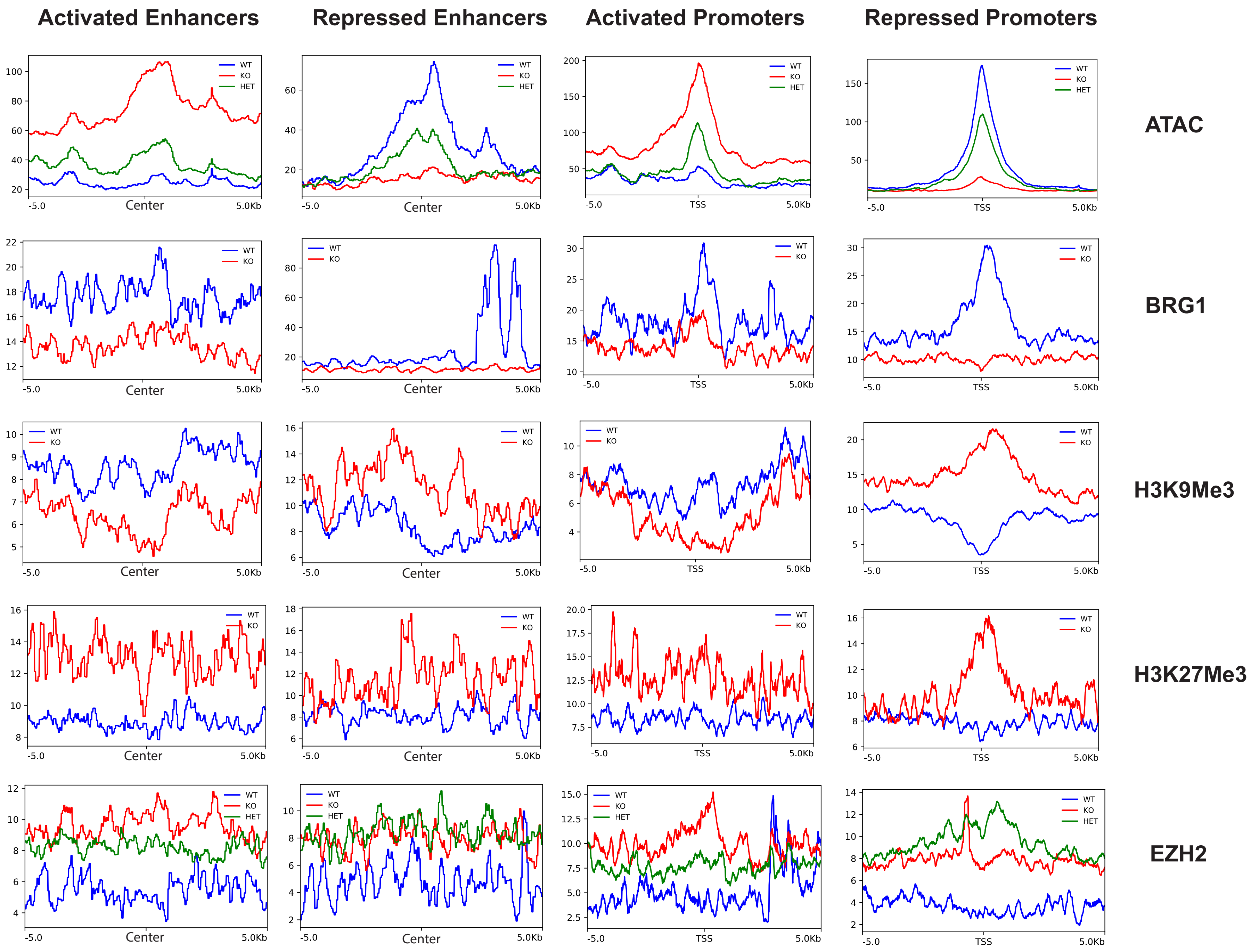

Supplementary Fig.1: ATAC and ChIP signal plotted in 10kb region surrounding center of enhancers and TSSs showing more than two-fold change in ATAC-signal FDR<0.05

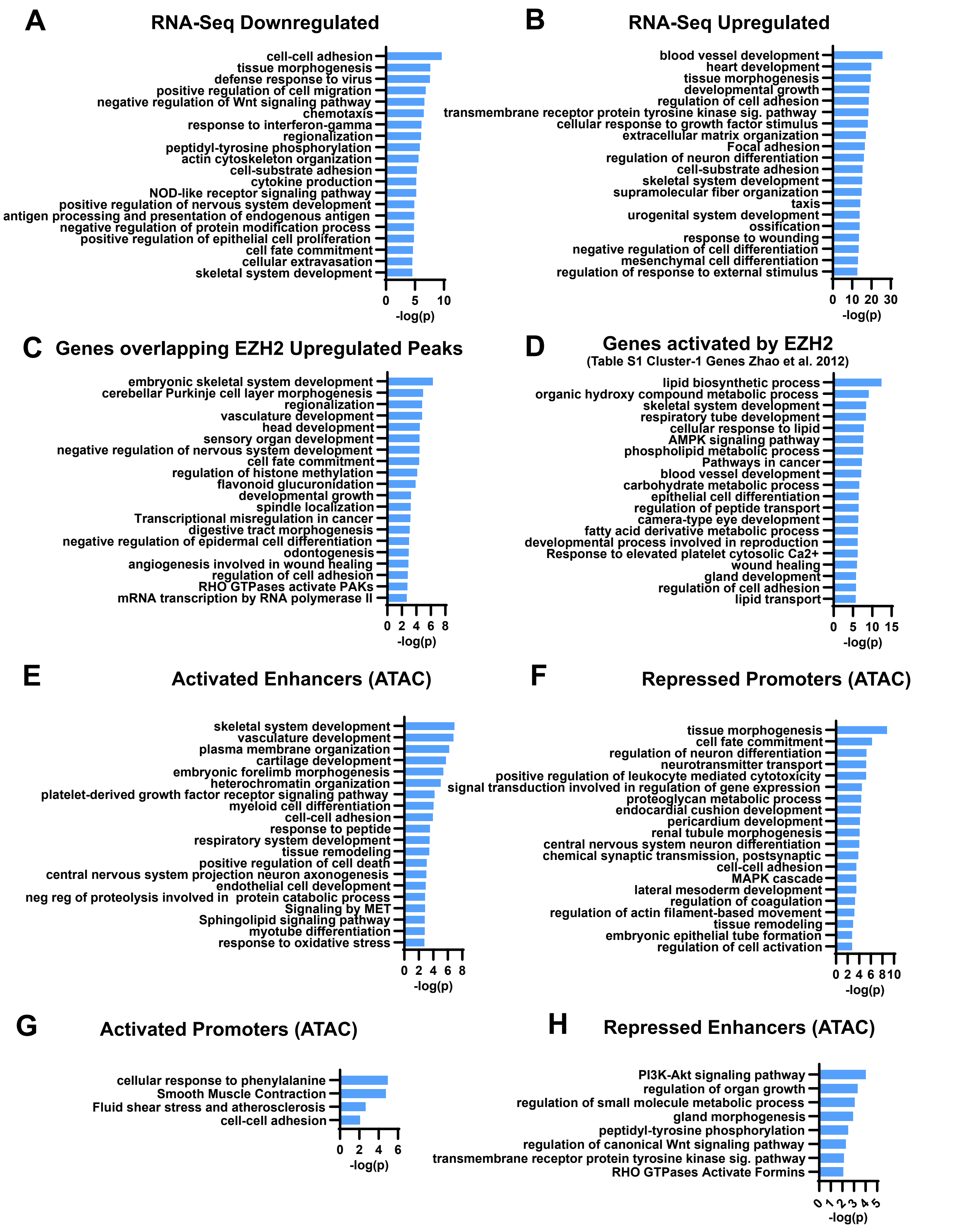

Supplementary Fig. 2: Top 20 GO-Terms associated with A) Genes downregulated by two-fold or more in KO cells with FDR<0.05 B) Genes upregulated by two-fold or more in KO cells with FDR<0.05 C) Genes overlapping EZH2 peaks gaining two-fold or more ChIP-Seq reads in KO cells D) Androgen-Induced genes reported to be upregulated by EZH2 (Zhao et al. Genome Res. 2012 Feb;22(2):322-31) E) TSSs linked to enhancers showing more than two-fold increase in ATAC-signal in KO cells FDR<0.05 F) Promoters showing more than two-fold decrease in ATAC-signal in KO cells FDR<0.05 G) Promoters showing more than two-fold decrease in ATAC-signal in KO cells FDR<0.05 H) TSSs linked to enhancers showing more than two-fold decrease in ATAC-signal FDR<0.05. p-values based on one-tailed hypergeometric test for all plots.

A

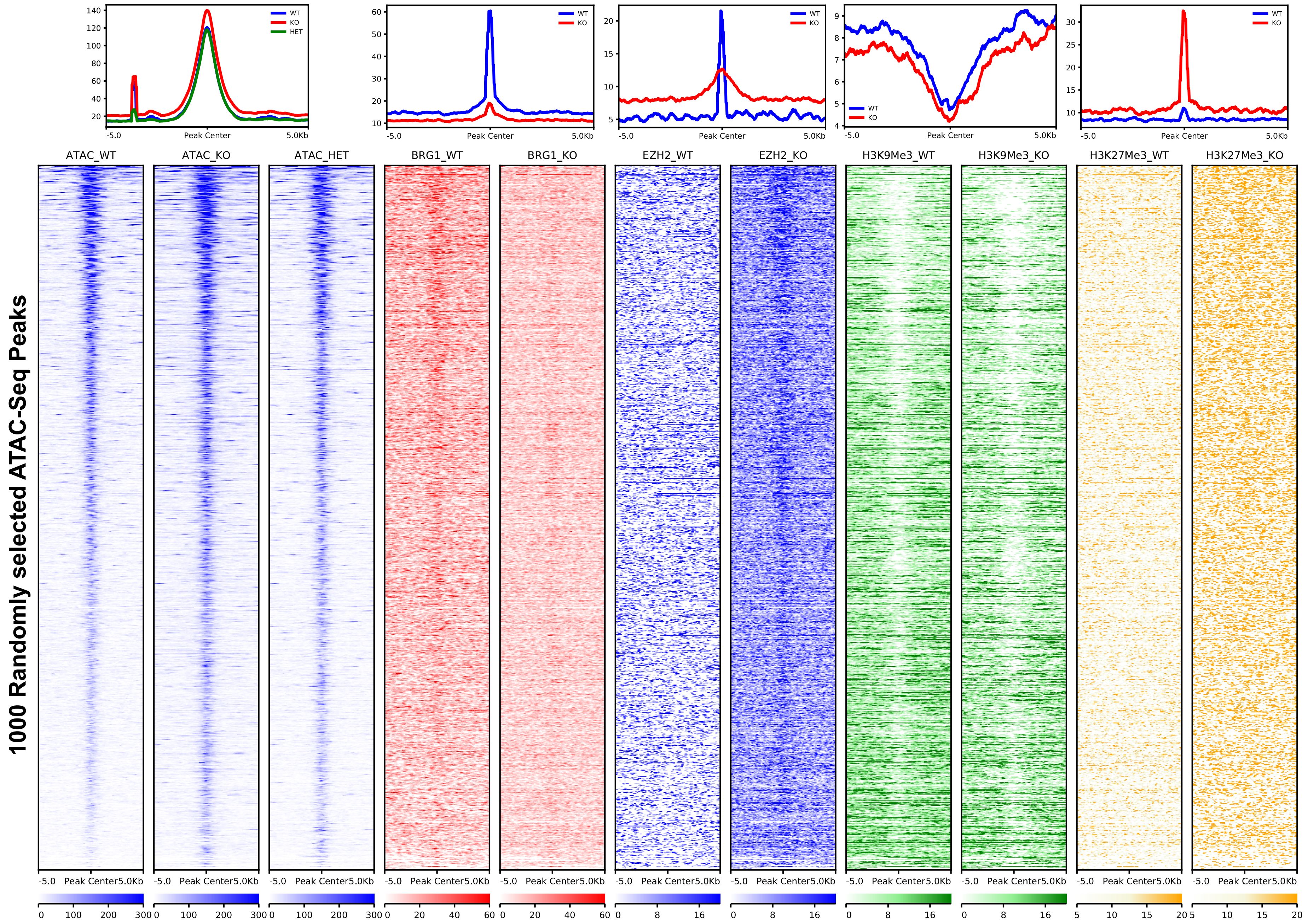

Supplementary Fig. 3: A) Density plots showing average signal intensities (top) and heatmaps displaying scaled read densities (bottom) for ATAC (blue), BRG1 (red), EZH2 (blue), H3K9me3 (green) and H3K27me3 (yellow) in regions ± 5 kb of 1000 random ATAC-Seq peaks. Plots are sorted by WT ATAC signal Scale bar shows normalized RPKM B) Nucleosome occupancy plots of 5kb regions surrounding all TSSs and randomly selected TSSs.

Supplementary Fig. 3 cont'd

B

Nucleosomal Signal

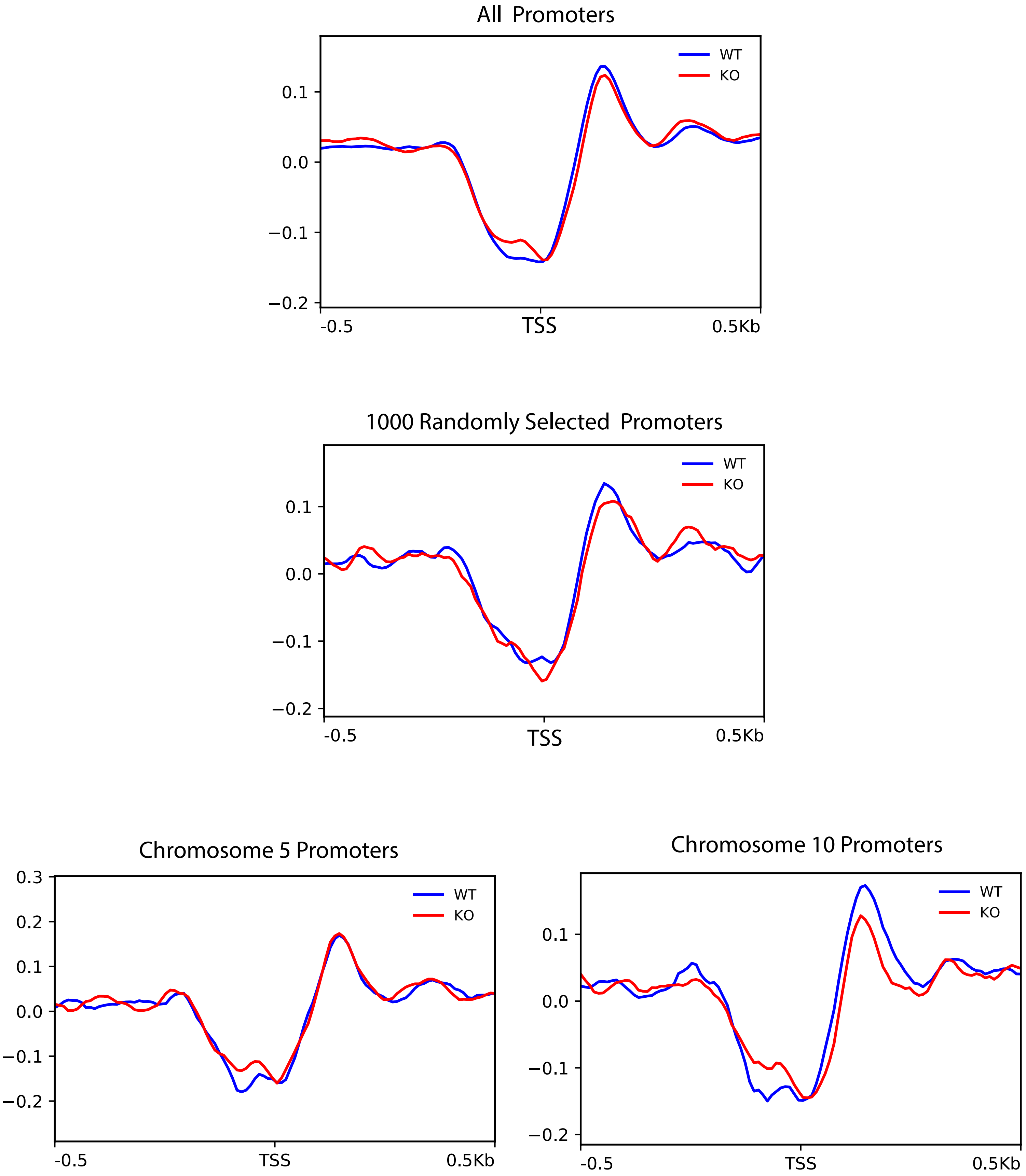

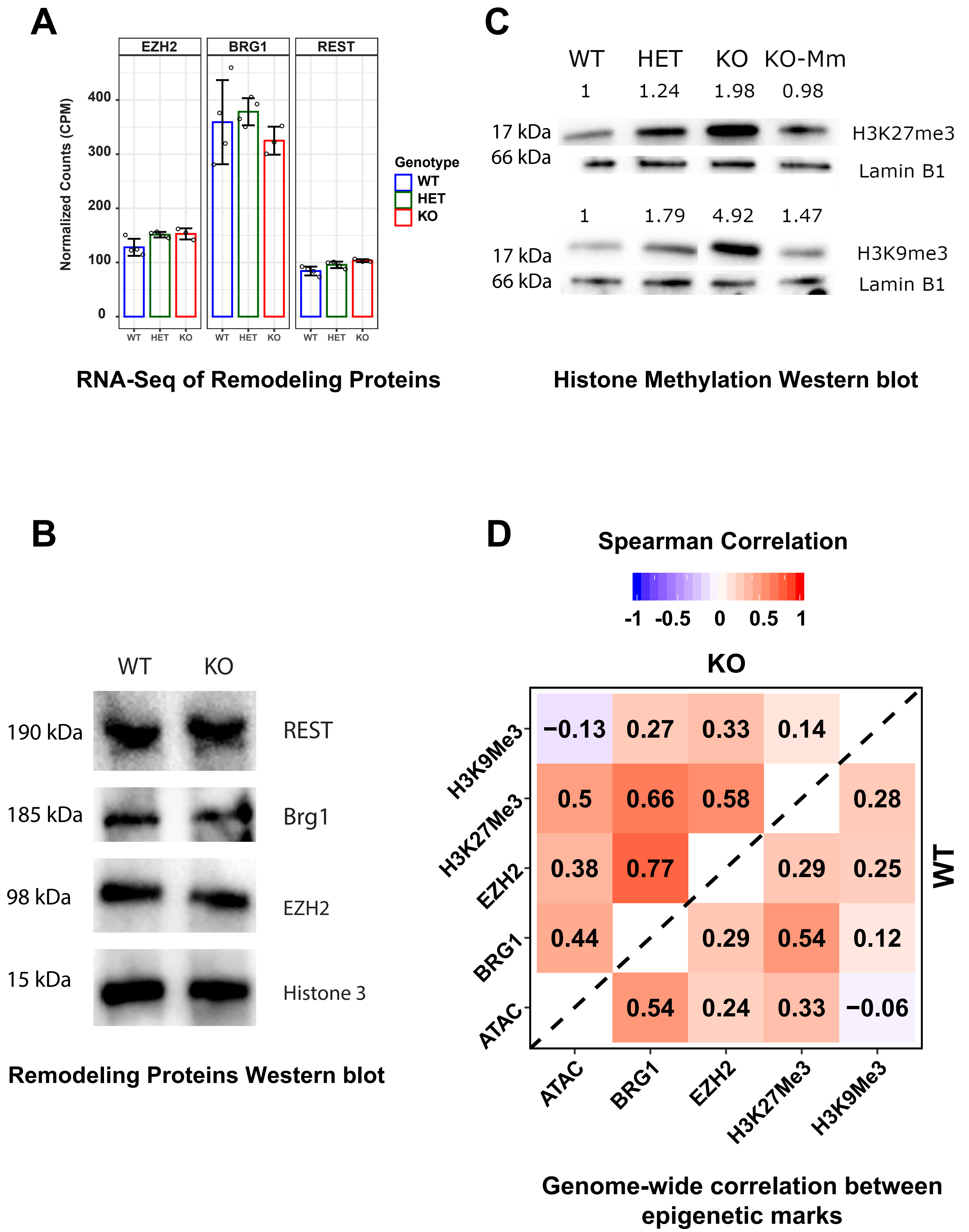

Supplementary Fig. 4: A) Normalized counts per million reads for EZH2, BRG1, and REST for wildtype, β-actin knockout and β-actin heterozygous MEFs. Error bars show mean ± sd. n= 4, 4 and 3 biological replicates for WT, HET and KO respectively B) Western blot analysis of REST/NRSF, Brg1, EZH2 expression level in the nuclear fraction of WT and KO cells, histone 3 is used as loading control. Results based on a single experiment C) Western blot analysis of H3K27me3 & H3K9me3 in WT, HET, KO & KO-Mm cells, Lamin B1 is used as loading control. Results based on a single experiment D) Genome-wide pairwise spearman correlation heatmap of all epigenetic marks in KO (top left triangle) and WT (bottom right triangle) cells

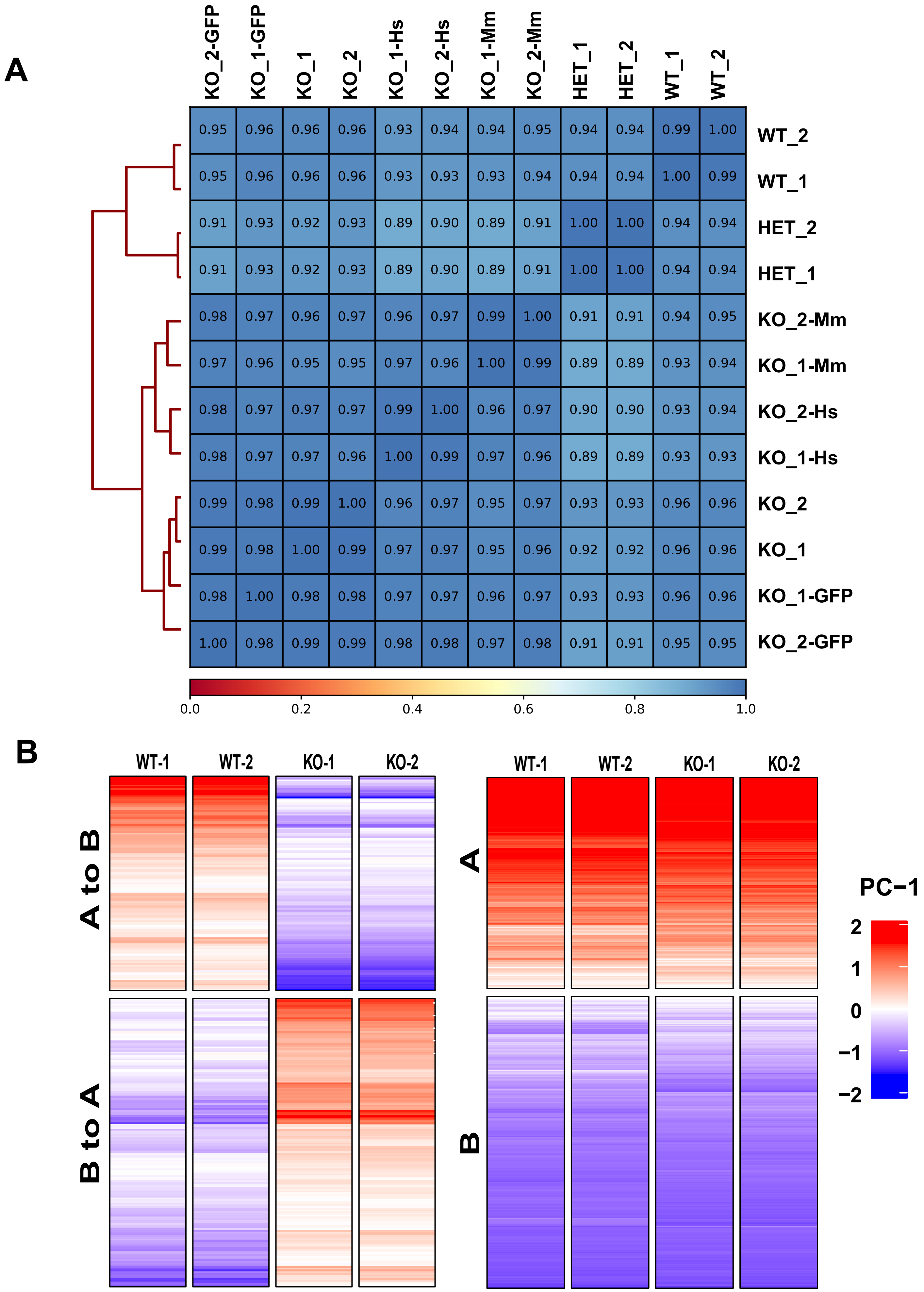

Supplementary Fig. 5: A) Pairwise spearman correlation heatmap based on aligned and filtered HiC reads (processed with HiCUP) showing correlation between replicates B) Heatmap of PC-1 values of 500kb bins switching from A to B or B to A showing high consistency between biological replicates Scalebar shows raw PC-1 value.

A ATAC tracks of selected gene promoters within switching compartments

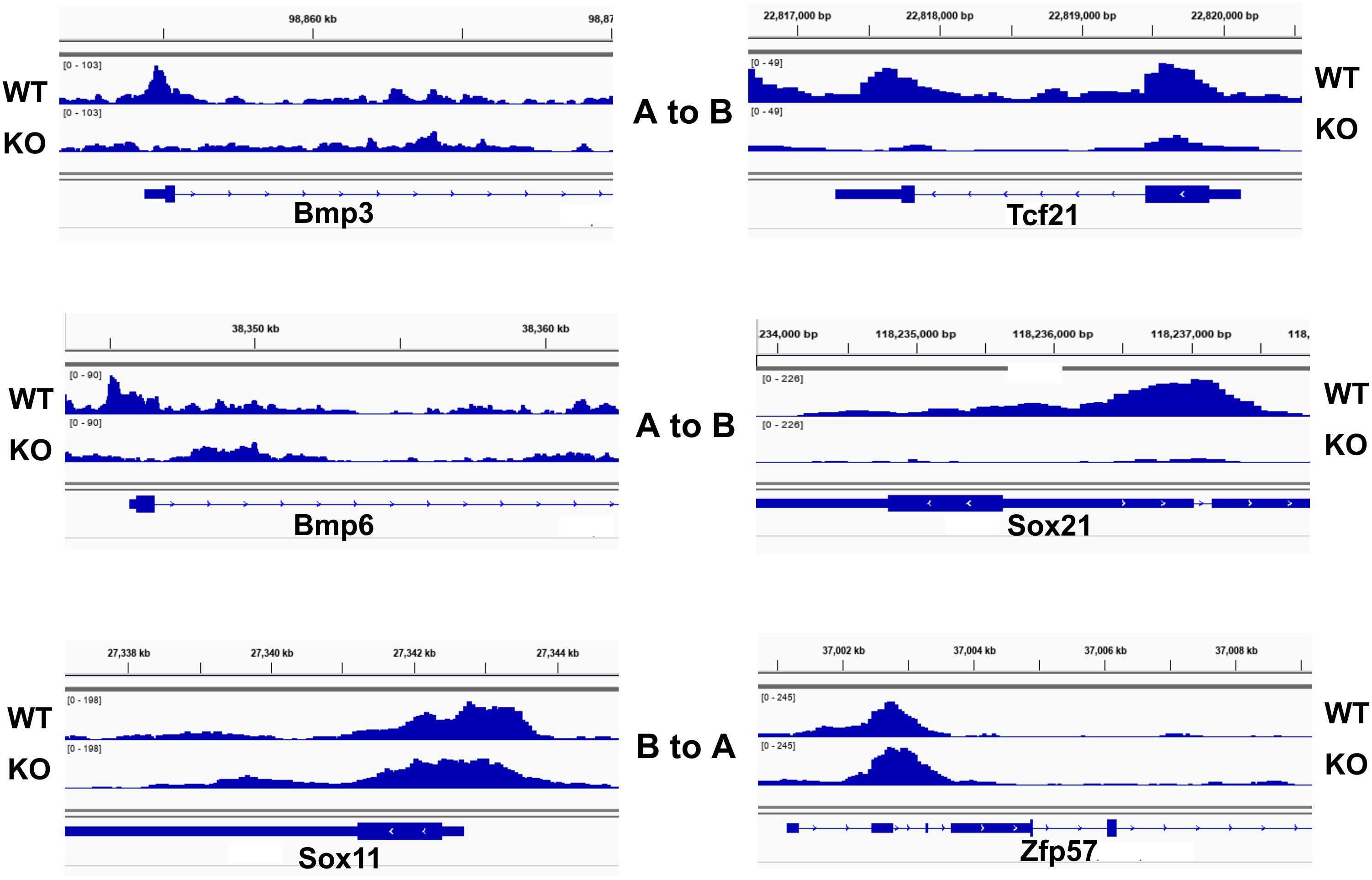

GO-Term Analysis

B

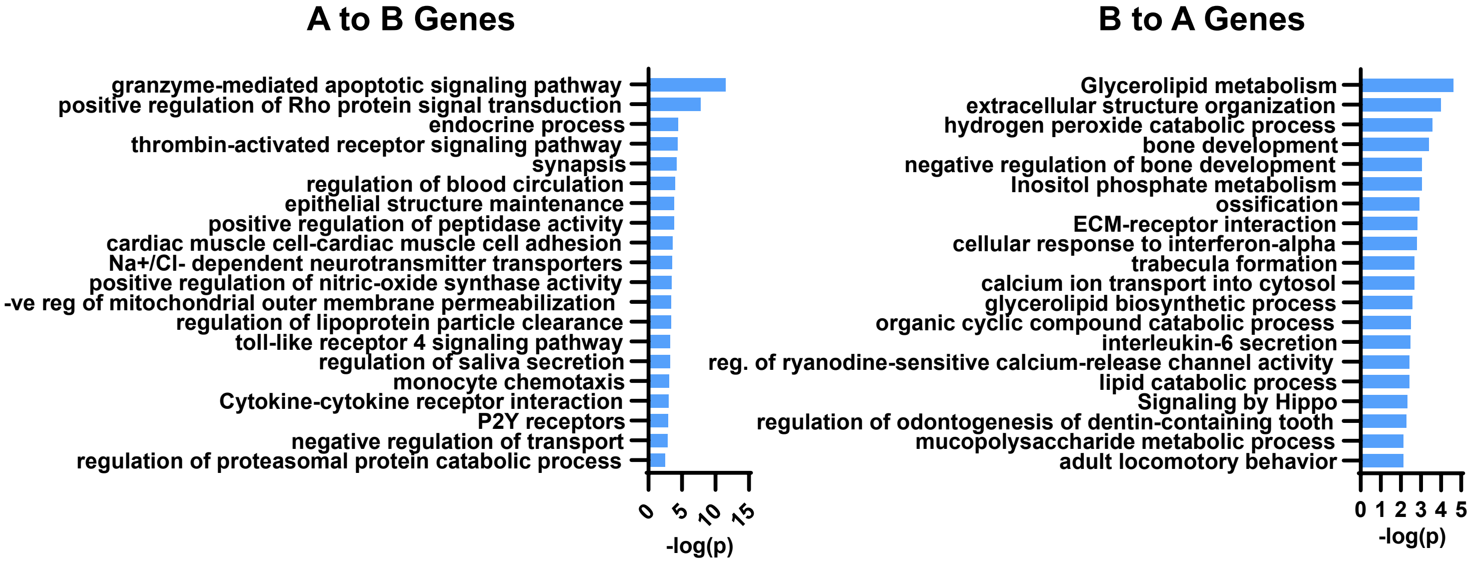

Supplementary Fig. 6: A) ATAC-tracks showing normalized signal (RPKM) at the promoters of select A to B and B to A switching genes B) Top 20 GO-Terms associated with protein-coding genes switching from to A to B (left) or B to A (right) compartments. p-values based on one-tailed hypergeometric test C) qPCR quantification of relative gene expression of selected switching genes. The Nono housekeeping gene was used for normalization and normalized WT expression was set to 1 for each gene. Biological replicates (n) are shown for each figure. Error bars show 95% confidence interval of mean. p-values based on two-tailed Welch t-test

Supplementary Fig. 6 cont'd

C

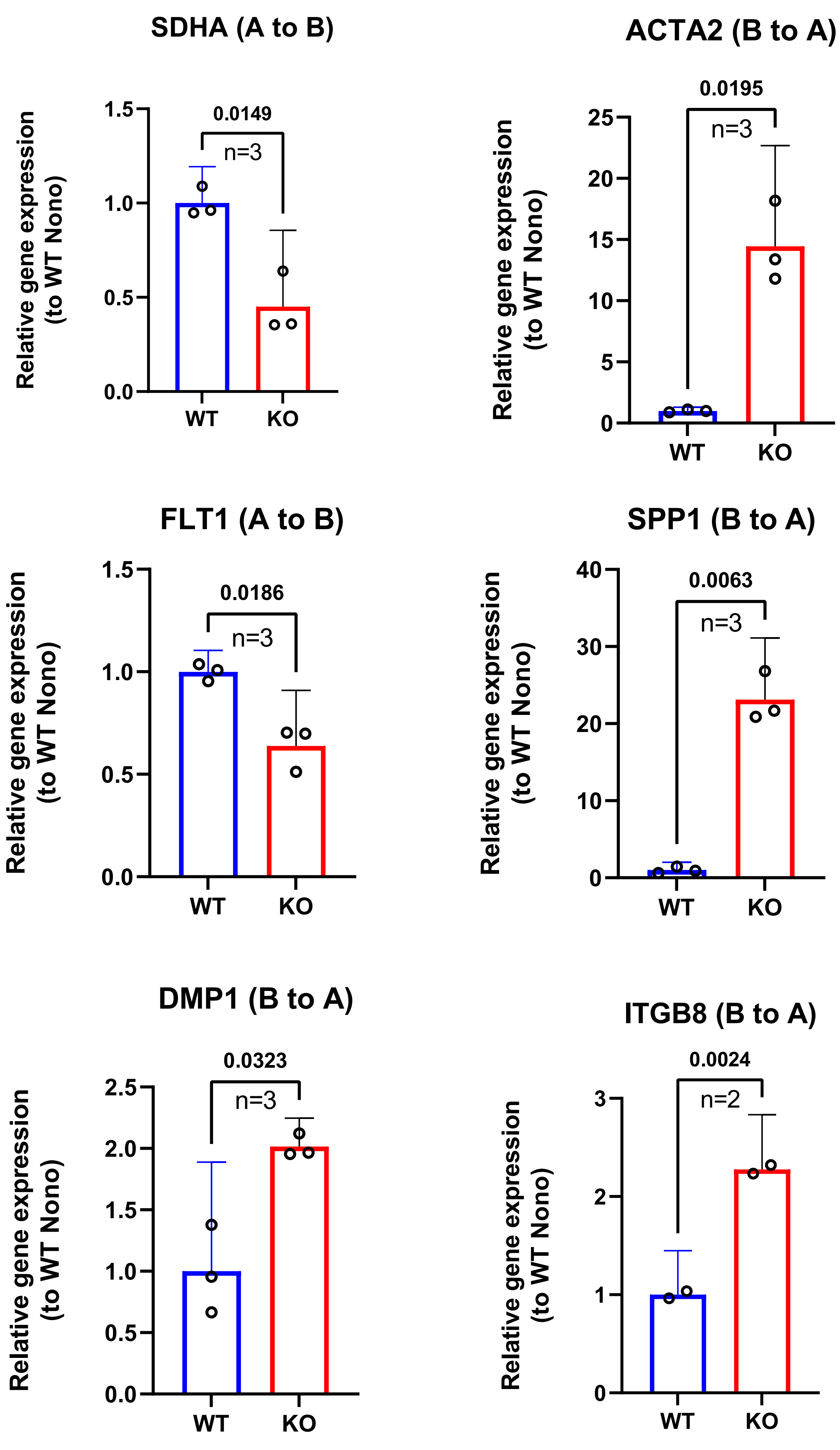

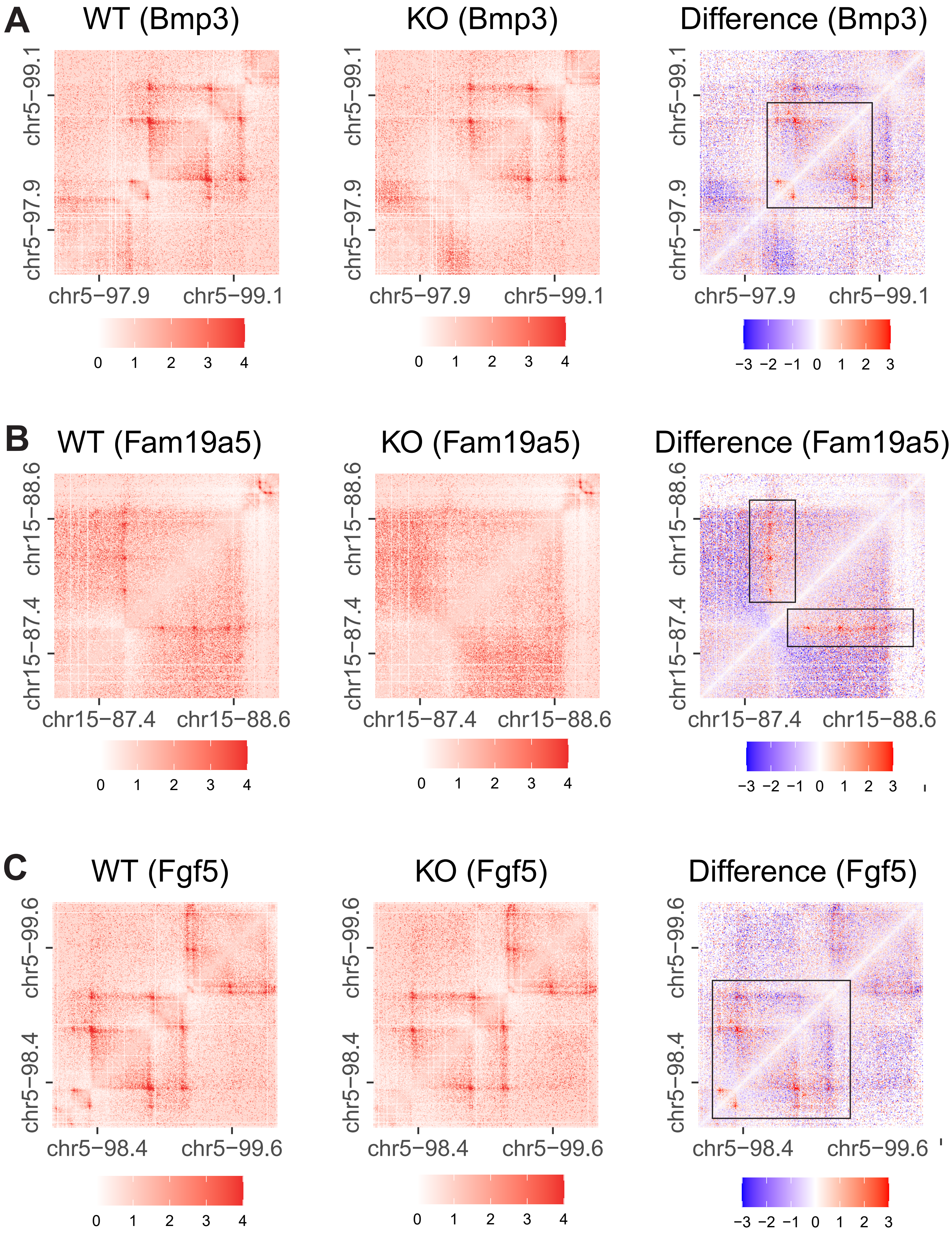

Supplementary Fig. 7: HiC interaction matrices at 4kb resolution showing 2bm regions of selected genes exhibiting differential interactions. Scalebar shows ratio of observed/expected interactions for WT and KO plots and WT minus KO ratio for difference plot. A) Bmp3 B) Fam19a5 C) Fgf5 D) Ndufaf2 E) Samd5 F) Sv2c

Supplementary Fig. 7 cont'd

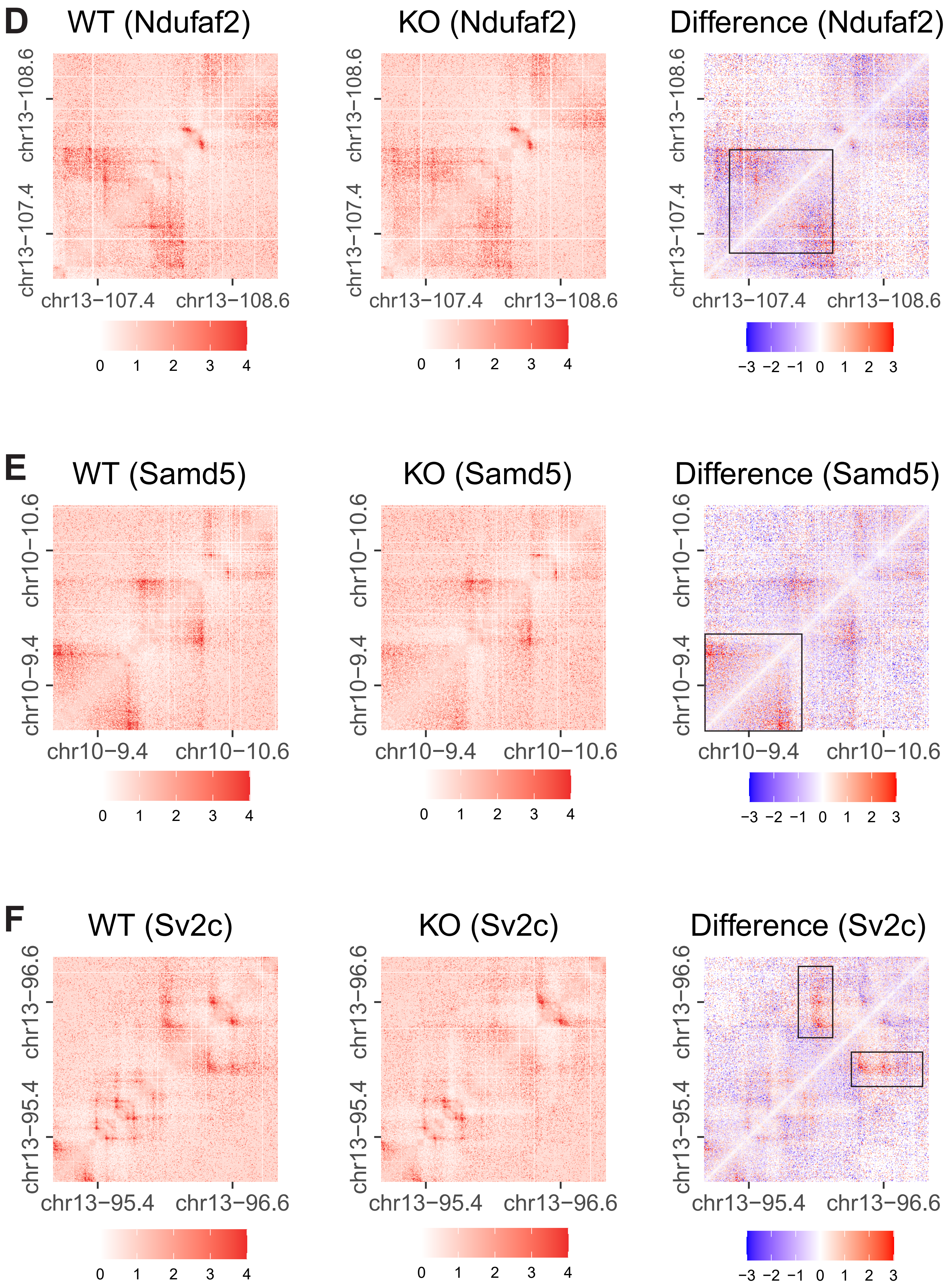

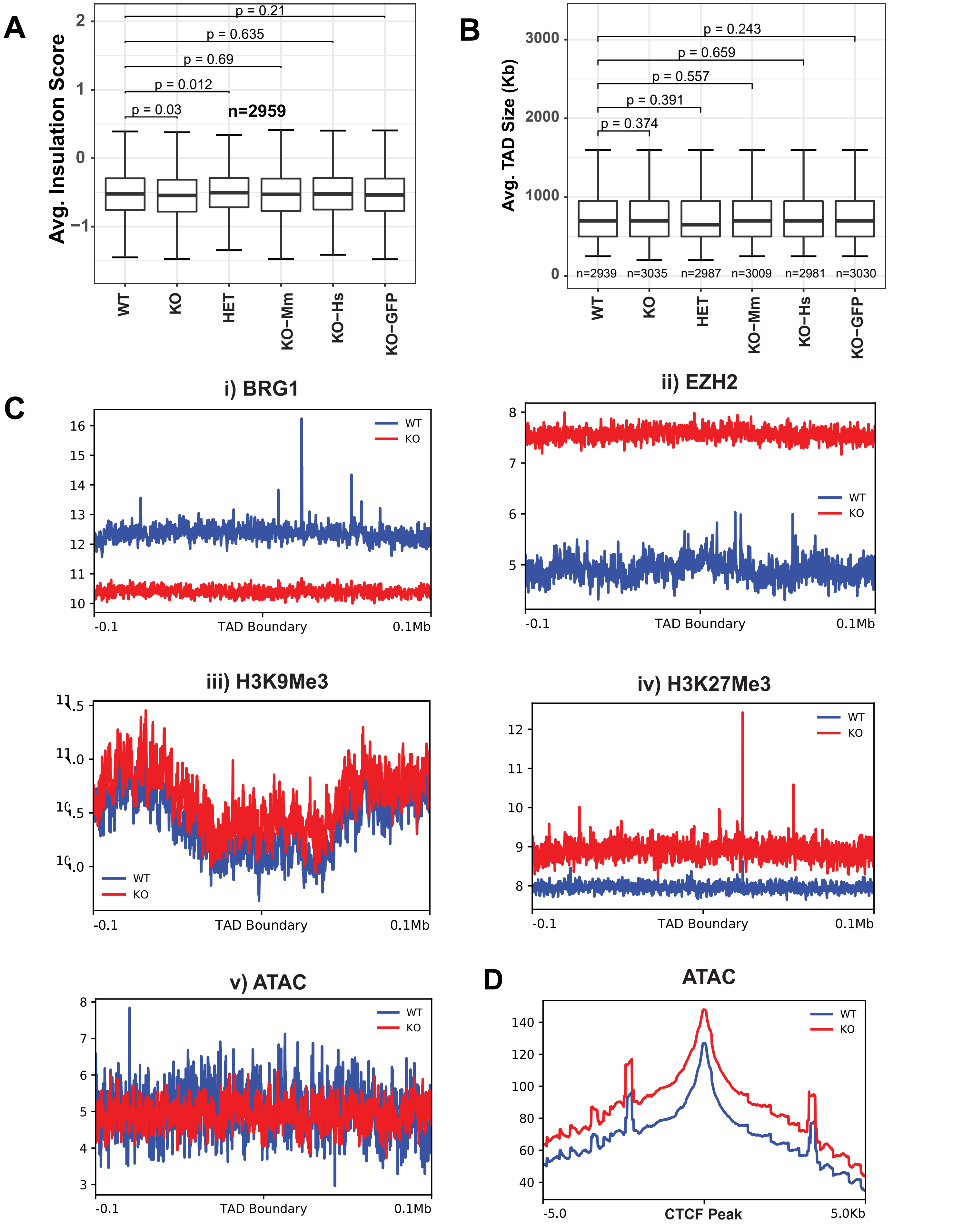

Supplementary Fig. 8: A) Boxplots of average insulation scores of all TAD boundaries. Boxes represent first and third quartiles with line in the box showing median and whiskers showing data within 1.5 x interquartile range. P-values based on two-tailed Wilcoxon rank sum test and adjusted for multiple testing using the Benjamini Hochberg procedure. n=Number of TAD boundaries B) Boxplots of average TAD size for all samples. Boxes represent first and third quartiles with line in the box showing median and whiskers showing data within 1.5 x interquartile range. P-values based on two-tailed Wilcoxon rank sum test and adjusted for multiple testing using the Benjamini Hochberg procedure. n=Number of TADs C) Average signal in RPKM in 200kb region surrounding center of TAD boundaries for i) BRG1 ii) EZH2 iii) H3K9Me3 iv) H3K27Me3 v) ATAC D) ATAC signal in RPKM in 10kb region surrounding ENCODE CTCF Peaks

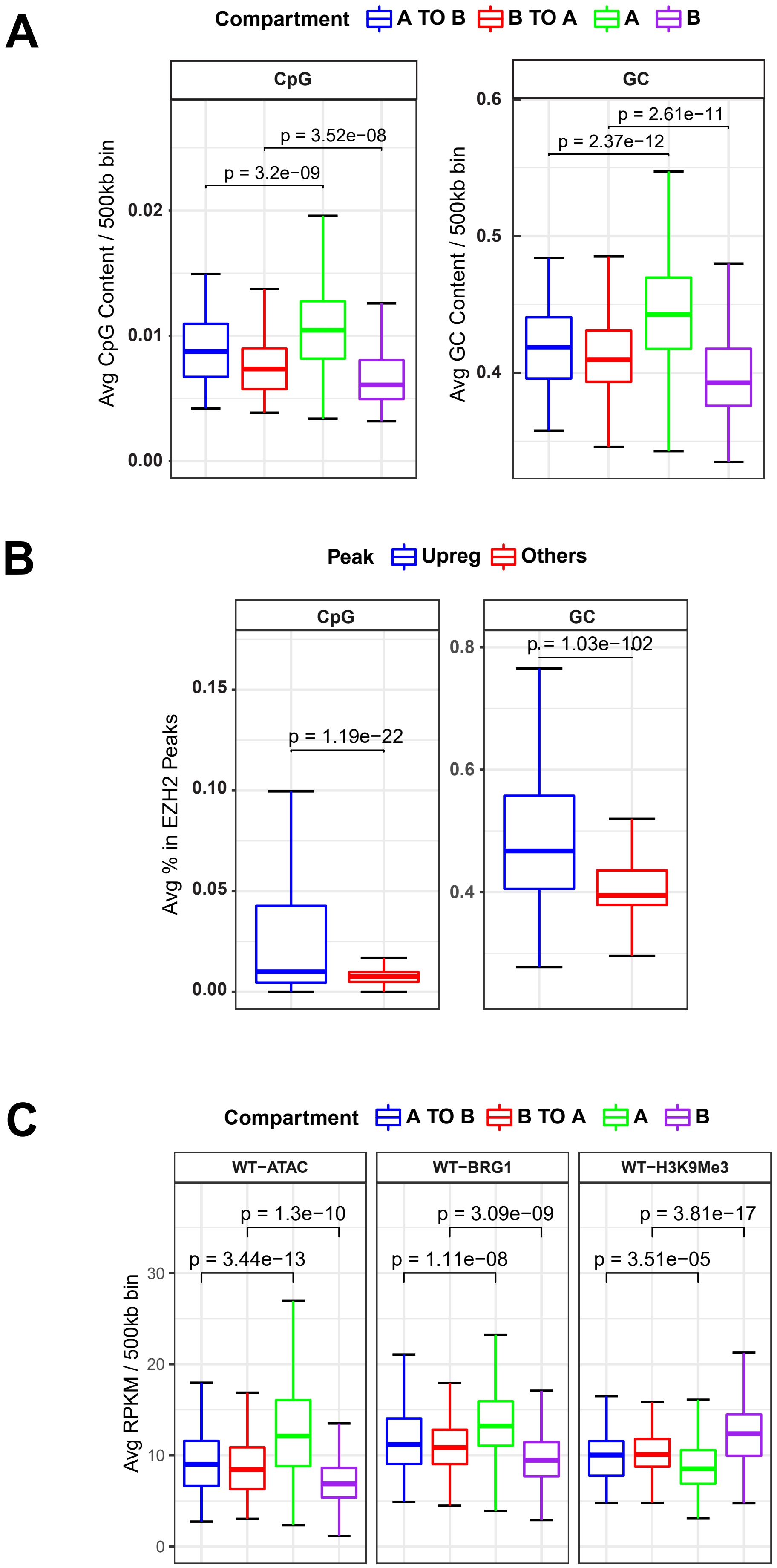

Supplementary Fig. 9: A) Average CpG (left) and GC (right) percentage for 500kb bins belonging to different compartments (n=125,168,1989 and 2749 for A to B, B to A, A and B compartments respectively). Average CpG (left) and GC (right) percentage in EZH2 peaks upregulated (n=928) in KO cells vs all other EZH2 peaks (n=12181). C) Average normalized counts (RPKM) per 500kb bin in each compartment type (n=125,168,1989 and 2749 for A to B, B to A, A and B compartments respectively) for i) ATAC, ii) H3K9Me3 iii) BRG1 in WT cells. For all plots, boxes represent first and third quartiles with line in the box showing median and whiskers showing data within 1.5 x interquartile range and p-values are based on two-tailed Wilcoxon rank sum test and adjusted for multiple testing using the Benjamini Hochberg procedure.

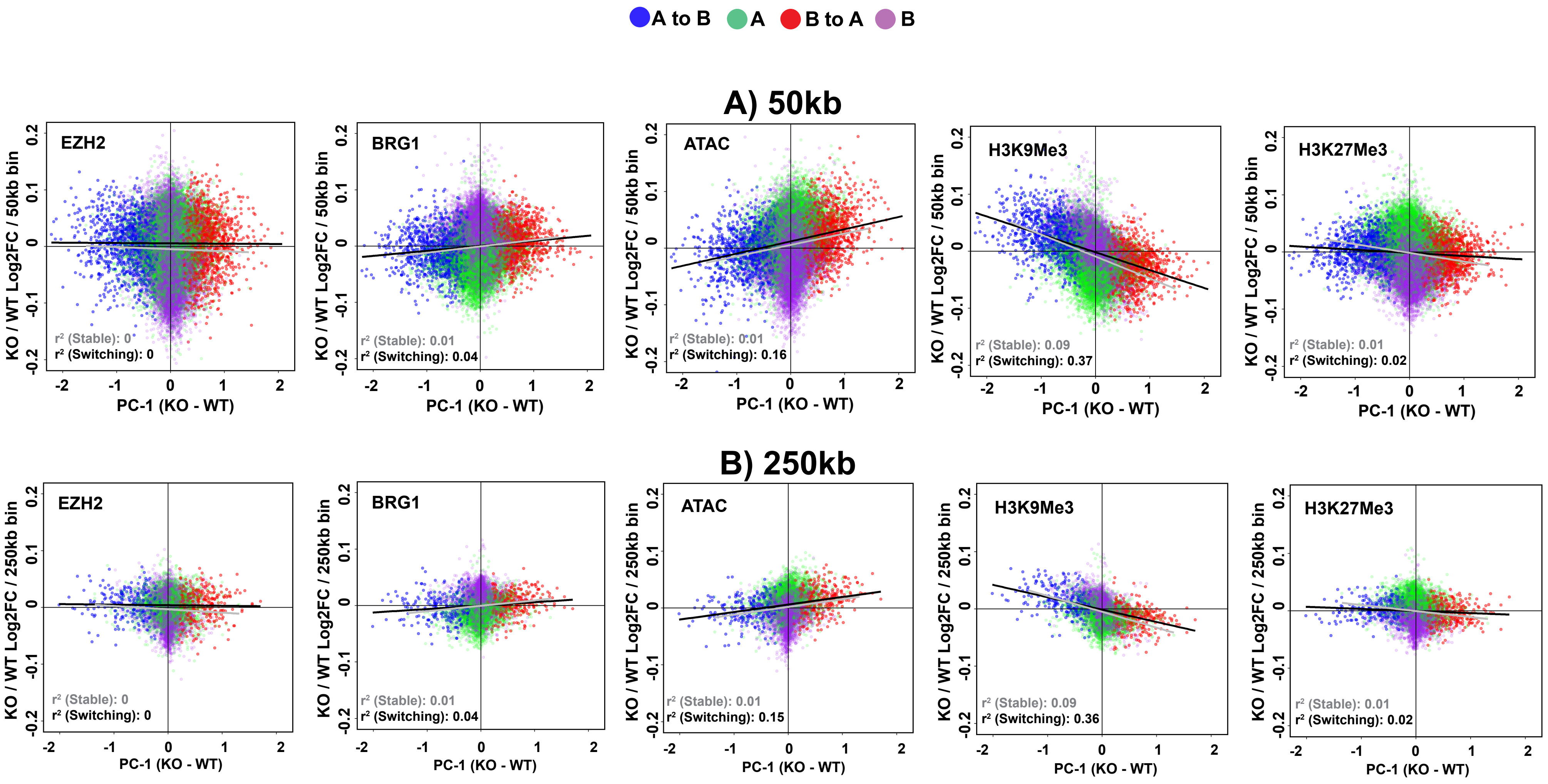

Supplementary Fig. 10: Scatterplots showing relationship between different epigenetic marks and compartment switching using a bin size of A) 50kb and B) 250kb. Scatterplots show KO over WT Log2FC in normalized counts for each genomic bin on the y-axis and difference between KO and WT PC-1 value for each genomic bin on the x-axis. R-Squared and line of best fit for switching bins only (A to B and B to A) shown in black, R-Squared and line of best fit for stable bins only (A and B) shown in grey.

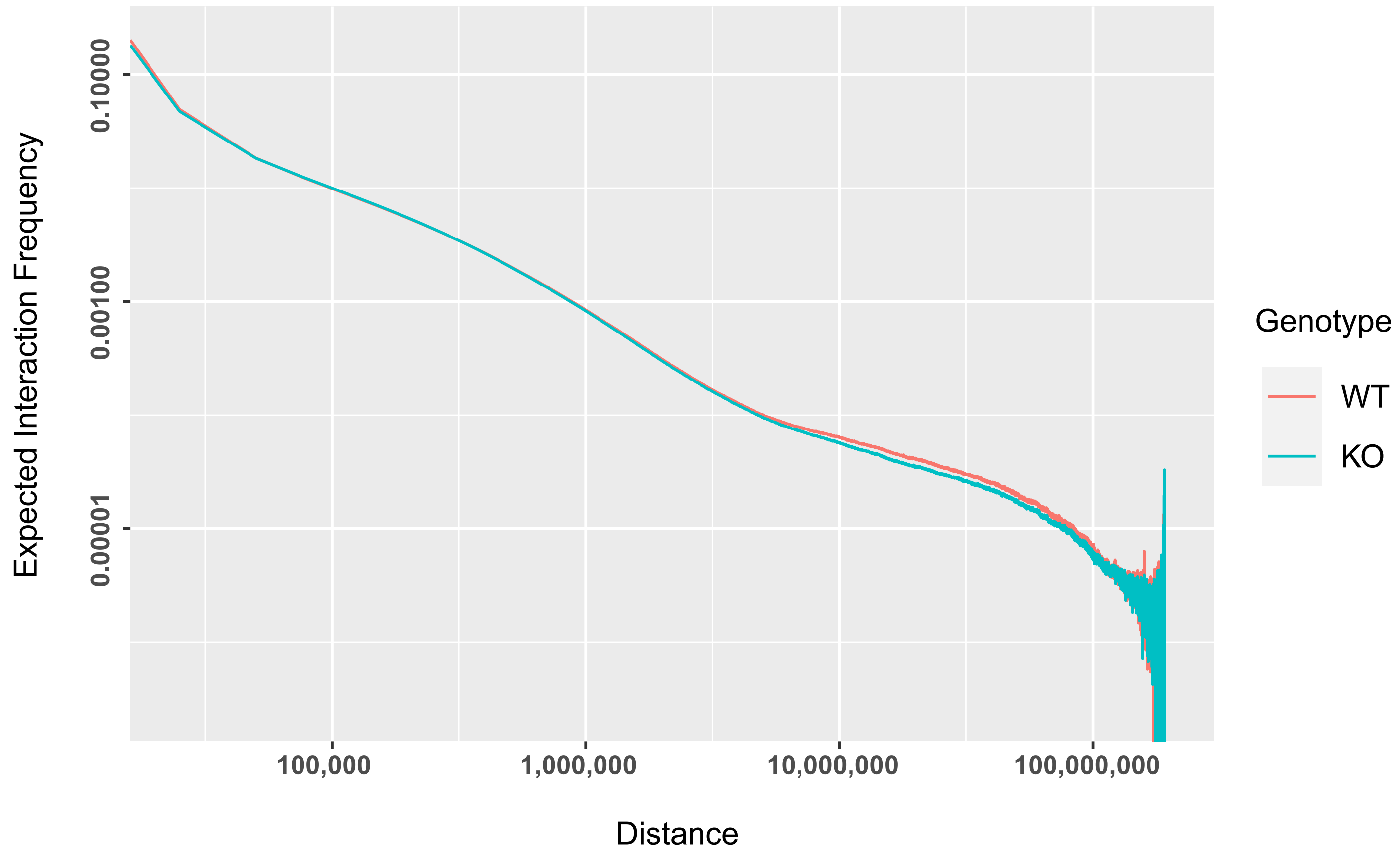

Supplementary Fig. 11: Expected interaction frequency as a function of genomic distance in WT and KO cells based on HOMER background model at 25kb resolution.

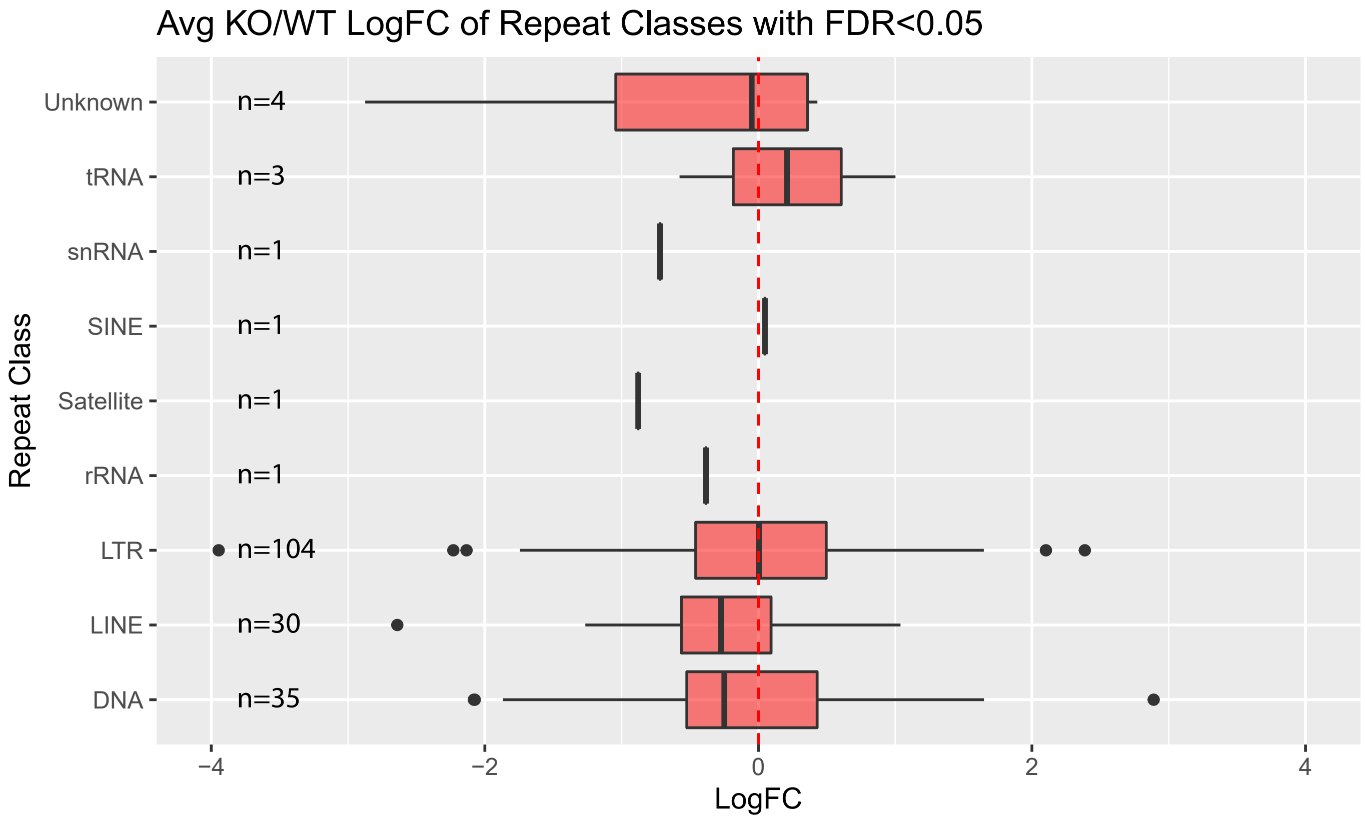

Supplementary Fig. 12: Boxplots showing differential expression of repeat element classes between WT and KO cells. Number of repeat classes in each category is shown to the left of each boxplot. Boxes represent first and third quartiles with line in the box showing median and whiskers showing data within 1.5 x interquartile range. n=Number of elements belonging to each class.

Supplementary Information

Supplementary Table 1

Hi-C quality control statistics						
Sample	Total Pairs	Valid_Pairs	Deduplicated_Uniques	Cis_Close (<10kbp)	Cis_Far (>10kbp)	Trans
KO_1_S3_L001_R1_2_001	309,644,365	189,950,111	71,104,689	17,154,650	47,289,438	6,660,601
KO_1_S3_L002_R1_2_001	320,826,500	196,769,315	72,420,974	17,465,070	48,174,545	6,781,359
KO_2_S9_L001_R1_2_001	329,860,889	196,844,135	72,701,436	18,522,338	47,031,393	7,147,705
KO_2_S9_L002_R1_2_001	342,475,566	204,372,204	74,185,867	18,897,604	47,994,826	7,293,437
WT_1_S1_L001_R1_2_001	205,026,071	125,398,474	47,612,393	11,088,878	31,874,004	4,649,511
WT_1_S1_L002_R1_2_001	213,373,821	130,498,921	48,666,647	11,325,629	32,592,402	4,748,616
WT_2_S7_L001_R1_2_001	452,366,919	246,438,160	84,596,901	23,442,266	53,183,429	7,971,206
WT_2_S7_L002_R1_2_001	471,340,281	256,756,558	86,352,619	23,917,007	54,303,485	8,132,127
KGN_1_S6_L001_R1_2_001	429,941,210	229,413,434	79,031,032	16,507,659	50,971,560	11,551,813
KGN_1_S6_L002_R1_2_001	444,906,432	237,523,519	80,383,105	16,788,548	51,853,558	11,740,999
KGN_2_S12_L001_R1_2_001	338,649,301	208,847,289	76,914,088	15,882,782	52,473,304	8,558,002
KGN_2_S12_L002_R1_2_001	353,590,853	218,025,463	78,854,887	16,282,154	53,806,889	8,765,844
KG_1_S4_L001_R1_2_001	256,818,180	160,564,354	60,736,764	12,080,435	42,281,655	6,374,674
KG_1_S4_L002_R1_2_001	265,215,633	165,782,472	61,741,394	12,280,142	42,981,876	6,479,376
KG_2_S10_L001_R1_2_001	462,857,204	282,782,385	103,014,066	21,048,546	70,366,185	11,599,335
KG_2_S10_L002_R1_2_001	479,234,820	292,754,458	104,875,888	21,425,235	71,647,591	11,803,062
Het_1_S2_L001_R1_2_001	328,258,853	165,732,487	55,992,011	16,399,458	34,220,696	5,371,857
Het_1_S2_L002_R1_2_001	337,387,964	170,289,593	56,682,144	16,610,084	34,634,455	5,437,605
Het_2_S8_L001_R1_2_001	416,535,494	215,898,833	74,313,236	21,652,663	45,785,800	6,874,773
Het_2_S8_L002_R1_2_001	430,563,412	223,122,655	75,492,557	21,995,121	46,512,398	6,985,038
KN_1_S5_L001_R1_2_001	398,962,537	219,508,043	75,774,683	16,058,982	49,506,475	10,209,226
KN_1_S5_L002_R1_2_001	413,159,348	227,539,991	77,204,508	16,364,445	50,449,758	10,390,305
KN_2_S11_L001_R1_2_001	397,616,123	245,952,847	92,636,500	19,649,607	62,892,003	10,094,890
KN_2_S11_L002_R1_2_001	411,106,210	254,277,629	94,254,589	19,985,024	63,995,731	10,273,834

Supplementary Table 2

Primer Sequences		
	Fwd Primer	Rev Primer
ITGB8	AGTGAACACAATAGATGTGGCTC	TTCCTGATCCACCTGAAACAAAA
FLT1	CACTGACATACCCAAACTTGTGC	GTCCCATGTTATTCTTTGCCCAT
ACTA2	GAGGCCTGGGTCTCTTCCA	GCTGAGCTGCCTCCTGTTTC
DMP1	CACGGACAGCAGTGAATCTGG	GCCGGTCCCCGTACTCTTA
SPP1	AGCAAGAAACTCTTCCAAGCAA	GTGAGATTCGTCAGATTCATCCG
SDHA	GGAACACTCCAAAAACAGACCT	CCACCACTGGGTATTGAGTAGAA
