# Supplementary material for: β-actin dependent chromatin remodeling mediates compartment level changes in 3D genome architecture
Source: Nat Commun. 2021 Sep 2;12:5240. doi: 10.1038/s41467-021-25596-2 (PMC8413440; doi:10.1038/s41467-021-25596-2)
Supplement: Supplementary file 4 — Source Data [file 41467_2021_25596_MOESM4_ESM.zip › SourceData/Mahmood et al 2021 - Unprocessed images.docx]

**Figure 1A**


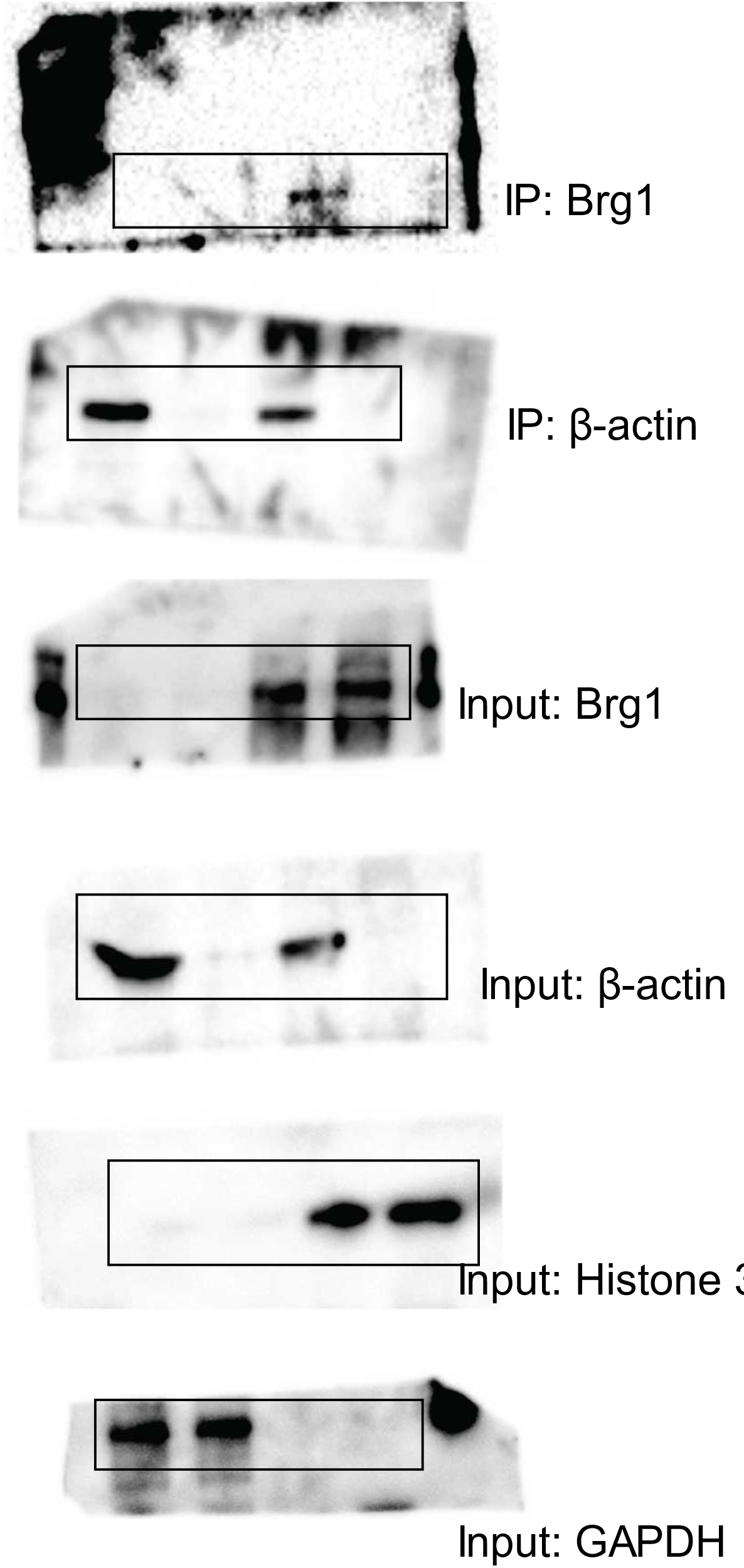


260 kDa

140 kDa

42 kDa

52 kDa

260 kDa

140 kDa

52 kDa

42 kDa

17 kDa

10 kDa

42 kDa

34 kDa

**Figure 1B**

52 kDa


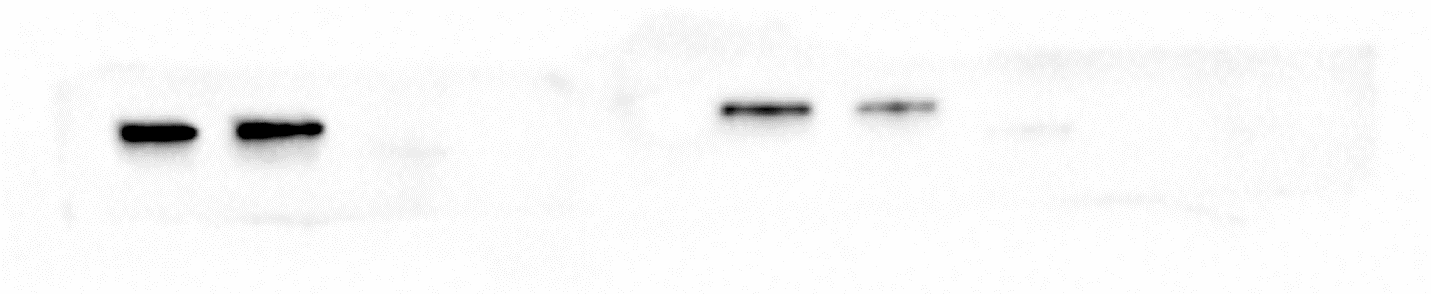


**β-actin**

42 kDa


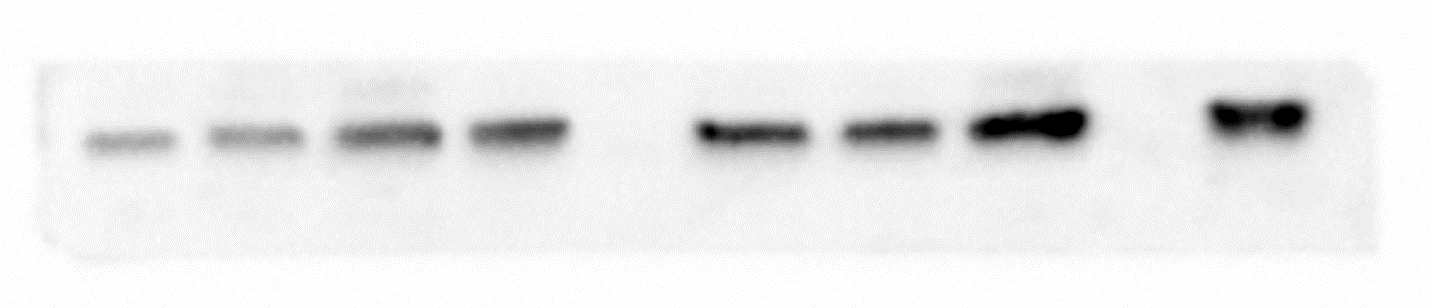


**Histone H3**

17 kDa

10 kDa


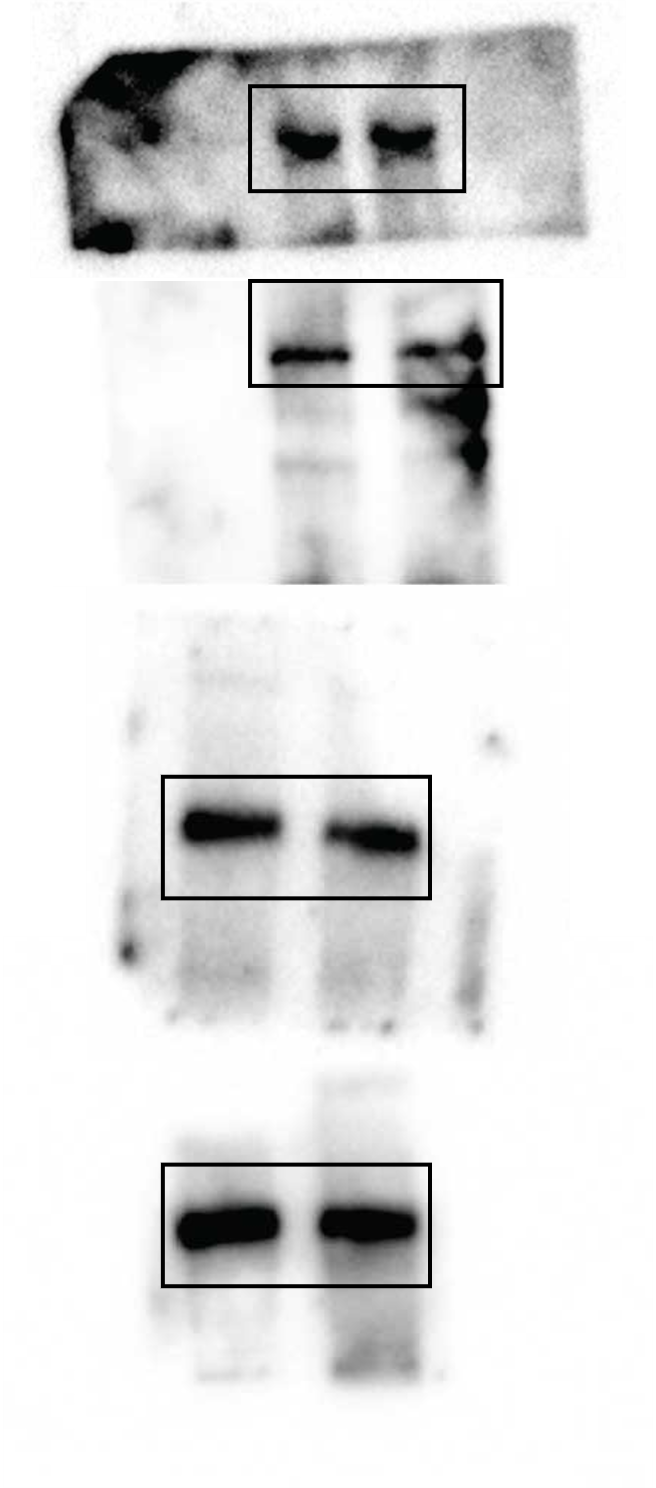
**Figure S4B**

260 kDa

**REST**

140 kDa

260 kDa

**BRG1**

72 kDa

140 kDa

**EZH2**

72 kDa

**Histone H3**

17 kDa

10 kDa

**
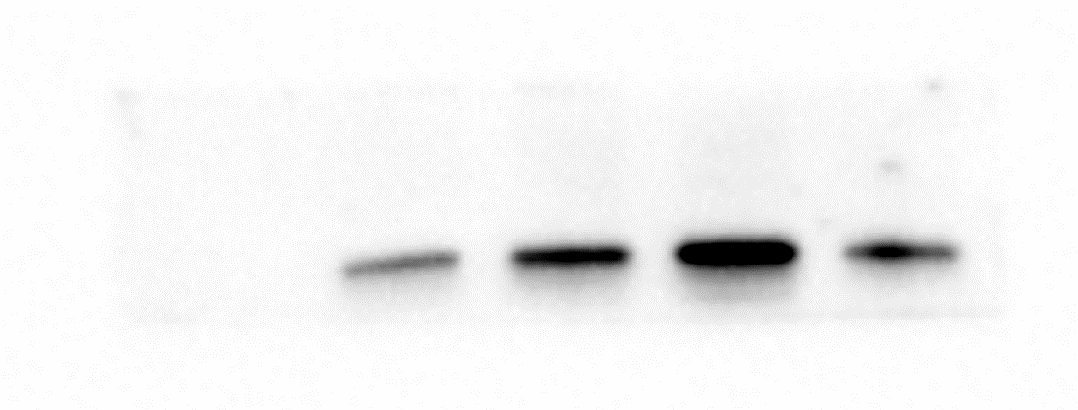
Figure S4C**

17 kDa

**H3K27me3**

10 kDa

**
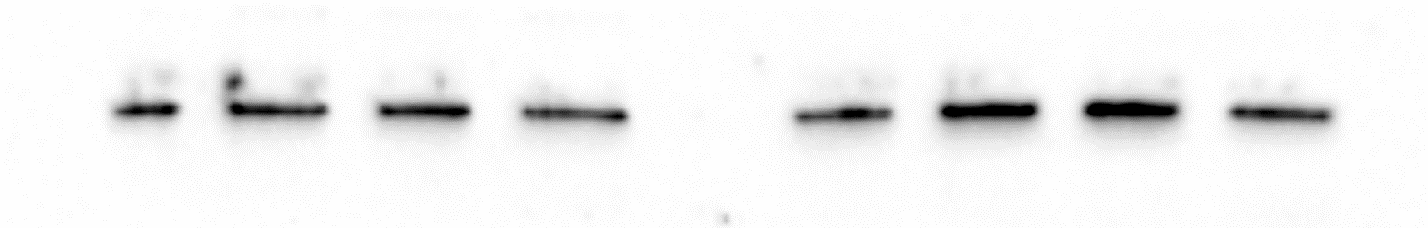
 Lamin B1**

52 kDa

72 kDa

**
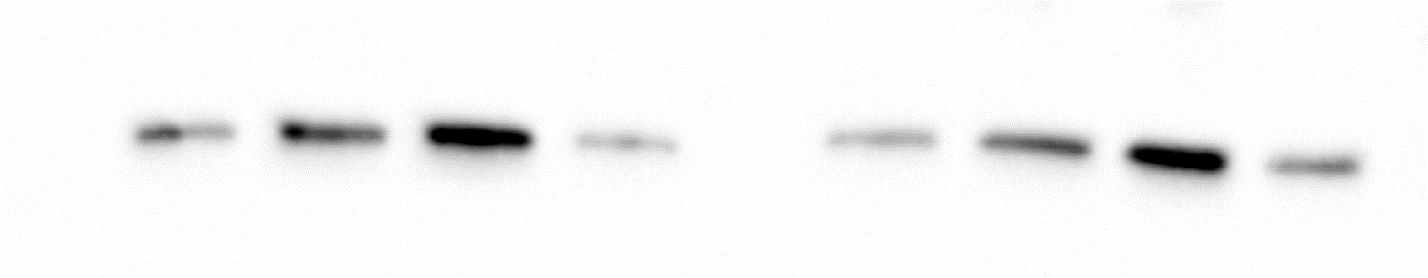
**

**H3k9me3**

17 kDa

10 kDa

**
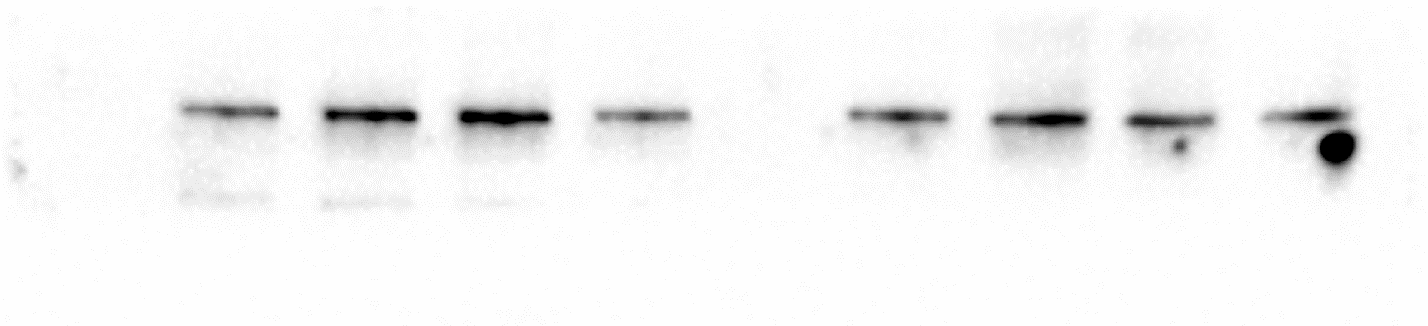
 Lamin B1**

72 kDa

52 kDa
